# The Use of Zwitterionic Methylmethacrylat Coated Silicone Inhibits Bacterial Adhesion and Biofilm Formation of *Staphylococcus aureus*

**DOI:** 10.3389/fbioe.2021.686192

**Published:** 2021-06-25

**Authors:** Franziska Woitschach, Marlen Kloss, Karsten Schlodder, Anne Rabes, Caroline Mörke, Stefan Oschatz, Volkmar Senz, Alexander Borck, Niels Grabow, Emil Christian Reisinger, Martina Sombetzki

**Affiliations:** ^1^Division of Tropical Medicine and Infectious Diseases, Center of Internal Medicine II, University Medical Center Rostock, Rostock, Germany; ^2^Biotronik, Berlin, Germany; ^3^Division of Cardiology, Center of Internal Medicine II, University Medical Center Rostock, Rostock, Germany; ^4^Institute for Biomedical Engineering, University Medical Center Rostock, Rostock, Germany

**Keywords:** biomaterials, biofilm, coating, 2-methacryloyloxyethyl phosphorylcholine, bacterial inhibition

## Abstract

In recent decades, biofilm-associated infections have become a major problem in many medical fields, leading to a high burden on patients and enormous costs for the healthcare system. Microbial infestations are caused by opportunistic pathogens which often enter the incision already during implantation. In the subsequently formed biofilm bacteria are protected from the hosts immune system and antibiotic action. Therefore, the development of modified, anti-microbial implant materials displays an indispensable task. Thermoplastic polyurethane (TPU) represents the state-of-the-art material in implant manufacturing. Due to the constantly growing areas of application and the associated necessary adjustments, the optimization of these materials is essential. In the present study, modified liquid silicone rubber (LSR) surfaces were compared with two of the most commonly used TPUs in terms of bacterial colonization and biofilm formation. The tests were conducted with the clinically relevant bacterial strains *Staphylococcus aureus* and *Staphylococcus epidermidis*. Crystal violet staining and scanning electron microscopy showed reduced adhesion of bacteria and thus biofilm formation on these new materials, suggesting that the investigated materials are promising candidates for implant manufacturing.

## Introduction

Bacterial contamination of cardiovascular implants is an increasing problem that can be fatal if left untreated. Complications due to biofilm on cardiovascular devices account for 4% in mechanical heart valves, 10% in ventricular shunts, 4% in pacemakers and defibrillator, and approximately 40% in ventricular-assisted devices (Darouiche, 2004). Therapeutic options range from specific drug/antibiotic therapy to removal and replacement of the implant (VanEpps and Younger, 2016).

Bacterial adhesion and the resulting biofilm formation on implants strongly depend on surface properties. Biofilms represent a multicellular community of bacteria that attach to solid surfaces and are surrounded by an exopolymer matrix. Bacteria in these aggregations are more resistant to antibiotic treatment and can evade the host’s immune defense (de La Fuente-Núñez et al., 2013; Lister and Horswill, 2014). Biofilm formation is the most common cause of catheter, pacemaker leads and heart valve infections (Peters et al., 1981; Marrie et al., 1982; Oliveira et al., 2018).

There are two main strategies to inhibit microbial colonization on surfaces: the use of materials with anti-microbial or anti-adhesive properties (Shi et al., 2008; Zhang et al., 2008; Lönn-Stensrud et al., 2009). An anti-microbial surface structure can be achieved by functionalization of the materials with bactericidal substances, such as incorporated or immobilized antibiotics. Anti-adhesive conditions can be achieved by the modification of the materials with anti-adhesive polymers (Kingshott et al., 2003). In the context of anti-adhesive polymers, polymethylmethacrylat (PMMA) has been investigated in various studies and is known as an appropriate biodegradable candidate for medical implants, e.g., as material for intraocular lenses, as bone filler or in the production of dental prostheses (Saha and Pal, 1984; Frazer et al., 2005; Tan et al., 2012; Muñoz et al., 2018). PMMA-based bone cement is used for minimally invasive stabilization of osteoporotic fractures to anchor artificial joints, such as hip and knee joints (Bistolfi et al., 2019). In addition, PMMA is routinely used as a base material for dental prostheses (Uzunoglu et al., 2014). Various attempts have been made to improve PMMA by modifying its surface with anti-bacterial substances such as chitosan or antibiotics, resulting in improved prevention of biofilm formation (Tan et al., 2012; Howlin et al., 2015). Optimization of PMMA by modification with phospholipid polymers or silver nanoparticles has shown to be beneficial regarding the prevention of biofilm formation (Tan et al., 2012; Shibata et al., 2016; Muñoz et al., 2018). PMMA modification with silver nanoparticles leads to a reduction in the formation of exopolymer subunits by >99% (Muñoz et al., 2018).

Surface modification by zwitterions displays another promising approach aiming at anti-adhesive features. Modifications containing zwitterions reduce the initial attachment of non-specific proteins to the material, which enable subsequent attachment of bacteria (Azari and Zou, 2013). One of the best characterized zwitterions is 2-methacryloyloxylethyl phosphorylcholine (MPC). MPC is a methacrylate with a phosphorylcholine group in the side chain that creates the zwitterionic structure by consisting of a phosphate anion and a trimethylammonium cation. MPC can reduce the adhesion of *E. coli* as much as 90% (Lewis, 2000). MPC-polymer treatment for acrylic resin material was shown to suppress *Candida albicans* adherence, non-*Candida albicans Candida* (NCAC), and methicillin-resistant *Staphylococcus aureus* (MRSA) via hydrophilic interactions between bacteria and surface (Fujiwara et al., 2020). The wide range of applications underlines the high biocompatibility and thus the useful application of PMMA in different medical settings. The surface modification of biomaterials with the aim of preventing implant-associated infections is an essential part of research. In this context, zwitterions, such as MPC, have great potential due to their anti-fouling properties. This anti-fouling effect is produced by an interplay of two mechanisms. The first effect is the formation of a hydration shell based on electrostatic interactions that hinders protein attachment to the surface. Secondly, steric hindrance is achieved by the zwitterionic polymer chains due to their hydrophilicity and mobility (Chen et al., 2010; He et al., 2016). A study by Wang et al. (2020) using MPC in combination with polyurethane (PU) described long-term inhibition of biofilm formation for *Pseudomonas aeruginosa* and *Staphylococcus epidermidis*. The advantages of non-fouling zwitterionic materials, such as PMMA-MPC, include simplicity of synthesis, ease of application, abundance of raw materials and availability of functional groups (Chen et al., 2010). Other examples of polymers with anti-adhesive character are polysulfonates (PSU) and poly(1,4-phenylene-ether-ether-sulfones) (PPSP), which are widely used in the manufacture of membranes for ultrafiltration (Yunos et al., 2014). The hydrophobic properties, high pH and temperature resistance also qualify these materials as antimicrobial. Moreover, the additional ether compounds of PPSP lead to improved mechanical and chemical properties (Maheswari et al., 2013).

In this study, two thermoplastic polyurethanes (TPU), unmodified and modified liquid silicone rubber (LSR) were compared. Both types of materials are widely used in the medical field. In order to adapt the properties of the LSR to the clinic-specific requirements in terms of better anti-microbial performance, three different polymer modifications were tested. We used (i) polymethylmethacrylate-2-methacryloyloxyethyl phosphorylcholine (PMMA-MPC), (ii) polysulfonate (PSU), and (iii) poly (1,4-phenylene-ether-ether-sulfones) (PPSP). The different material surfaces were examined with regard to their tendency to bacterial colonization and biofilm formation by *Staphylococcus aureus* and *Staphylococcus epidermidis*. Both bacterial strains are frequently associated with medical devices infections (de La Fuente-Núñez et al., 2013), most common cause involved in orthopedic implant-associated infections (Li and Webster, 2018) and known as causative triggers for infectious endo- and myocarditis (Moreillon and Que, 2004; Sagar et al., 2012; Murillo et al., 2016). Despite their wide application as medical biomaterials, the use of (i) PMMA-MPC, (ii) PSU, and (iii) PPSP is not established for cardiovascular implants so far. Considering the age of antibiotic resistance, it is highly relevant to explore innovative strategies for bacterial defense.

## Materials and Methods

### Material Functionalization

Two different implant materials, a TPU and a platinum-cured LSR, were selected as target materials for the comparative analysis. TPU samples were produced by extruding a film from the TPU granulate using a twin-screw extruder with a flat film nozzle. Round samples with a diameter of 16 mm were cut out. Two TPUs, TPU 55 and TPU 80 were used, differing in terms of material strength, where 55 indicates a greater hardness than 80. For processing of the LSR base material, the polymer was filled in a flat mold and vulcanized by a hot press. After a post-curing process in an oven, round samples with a diameter of 16 mm were cut out. The samples were differently coated.

#### PMMA-MPC Coating

LSR samples were swollen and treated with methyl methacrylate (MMA), methacrylic acid, azoisobutyronitril (AIBN), and methacryloyloxyethyl phosphorylcholine (MPC). Afterward the samples were rinsed with VE water.

#### PSU Coating

LSR samples were treated with PSU-DMAC (dimethy- lacetamid) solution.

#### PPSP Coating

**FIGURE 1:**
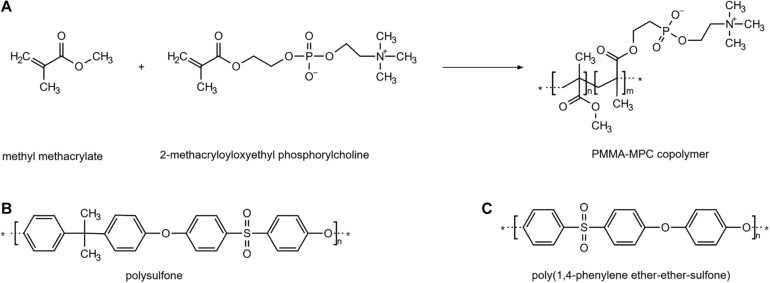
Functionalization of the material. Chemical structures of the molecules for the modification of the liquid silicone rubber (LSR). The asterisk marks the point at which the monomers combine. They represent a repetition unit. **(A)** polymethylmethacrylat+ 2-methacryloyloxyethyl phosphorylcholine (PMMA + MPC) **(B)** polysulfone (PSU) **(C)** poly(1,4-phenylene ether-ether-sulfone) (PPSP).

LSR samples were treated with PPSP-DMAC solution. After coating, all samples were cured in a drying oven, washed in a surfactant solution, and underwent a sterilization process with ethylene oxide (ETO). The chemical structures of the modifications (i–iii) are depicted in Figure 1. All known material specifications and mechanical characterization are summarized in Table 1. Round samples of thermanox, a well-established polyester, were used as reference material. In the following, abbreviations are used as follows: thermanox = tmx, TPU 55 = P55, TPU 80 = P80, unmodified LSR = LSR, LSR + PMMA-MPC = PMMA-MPC, LSR + PSU = PSU and LSR + PPSP = PPSP.

**TABLE 1:** Material specification and mechanical properties.

		Shore-hardness	Tensile strength in MPa	Elongation at break in %	Tear strength in kN/m
TPU	Thermoplastic polyurethane				
P55	Polyurethane 55	53D	44.8	450	105
P80	Polyurtehane 80	85A	28.9	650	73.6
LSR	Liquid silicone rubber				
LSR	Unmodified	65A	8.6	1,000	47.7
PMMA-MPC	Polymethylmethacrylate-2-methacryloyloxyethyl phosphorylcholine	65A	8.6	1,000	47.7
PSU	Polysulfone	65A	8.6	1,000	47.7
PPSP	Poly (1,4-phenylene-ehter-ether-sulfone)	65A	8.6	1,000	47.7
tmx	Thermanox	n.a.	n.a.	n.a.	n.a.

### Material Surface Characterization

Contact angle measurements were performed using a mobile surface analyzer MSA (Krüss GmbH, Germany) equipped by a double pressure dosing unit for hydrophobicity determination of the surface. Measurements were performed with 2 μl drop volume and 1 s equilibration time under constant conditions. Deionized water and diiodomethane (CH_2_I_2_) served as test liquids with different polarities to calculate the surface energy according to the OWRK (Owens, Wendt, Rabel, and Kaelble) method (Owens and Wendt, 1969; Kaelble, 1970). Contact angles were assessed in duplicates for each sample and calculated by averaging the values of both drop sides. Surface free energy (SFE) as well as polar and dispersive components were calculated using Krüss Advance software v. 1.9.2.

### Bacterial Strains and Culture Conditions

Snap frozen *Staphylococcus (S.) aureus* (ATCC35556) and *Staphylococcus (S.) epidermidis* (ATCC35984) were purchased from the American Type Culture Collection (ATCC). *S. aureus* was cultivated in Luria broth (LB) according to the supplier’s instructions and *S. epidermidis* was cultivated in tryptic soy broth (TSB). To ensure robust biofilm formation, media were supplemented with 1 or 0.25% glucose, respectively (Kwasny and Opperman, 2010). For all experiments the inoculum of each strain was prepared by adjusting the concentration of an overnight bacterial broth culture to 1 × 10^8^ CFU/ml (colony forming unit/ml) in LB or TSB medium corresponding to an OD_600_ of 0.2. All experiments were carried out aerobically at 37°C with 5% CO_2_ in 24-well polystyrene plates with a culture volume of 2 ml unless stated (Nunc, Thermo Fisher Scientific, Germany) and were carried out in three independent replicates.

### Bacterial Cell Surface Hydrophobicity

Hydrophobicity was assessed by microbial adherence to n-hexadecane in the MATH/BATH (Microbial Adhesion to Hydrocarbons/Bacterial Adherence to Hydrocarbons) test according to Lather et al. (2016) and Di Ciccio et al. (2015), with slight modification. Briefly, cells were harvested by centrifugation at 3,000 g for 5 min, washed three times in ice-cold phosphate buffer, and finally resuspended in phosphate buffer to achieve an OD500 of 0.5. The bacterial suspension was overlayed with 0.5 mL of n-hexadecane (Sigma Aldrich). After 1 min agitation by vortexing, the phases were separated for 15 min at room temperature. The results were expressed as the percentage of the cells excluded from the aqueous phase, determined by the equation as follows: % adherence = [(1 -A/A0)] × 100, with A0 and A as initial and final optical densities of the aqueous phase, respectively. The strains were classified as: highly hydrophobic, for values >50%; moderately hydrophobic, for values ranging from 20 to 50% and hydrophilic, for values <20%. The measurement was performed twice with two independent and three technical replicates.

### Bacterial Viability

Bacterial viability was assessed using water-soluble tetrazolium (WST, Microbial Viability Assay Kit-WST, GERBU Biotechnik, Germany) according to the manufacturer’s instructions. In brief, 1 × 10^8^ CFU were added to a 24-well plate containing the different rounded material disks with a diameter of 16 mm which were kindly provided from Biotronik (Berlin) and incubated for 1 h. The coloring reagent was added and after 2 h of incubation the absorbance was measured at 450 nm using a microplate reader (FLUOstar Omega, BMG Labtech). Tmx was used as reference and normalized to 100%.

### Bacterial Colonization

The ability of bacteria to adhere to the different material surfaces was analyzed using scanning electron microscopy (SEM). 2 × 10^8^ CFU were added to a 24-well plate containing the different material disks and incubated for 3, 7, 24, and 48 h. After incubation, each well was gently washed in 0.1 M phosphate buffer (PBS) to remove planktonic germs. The adherent bacteria were fixed with 2.5% buffered glutaraldehyde at 4°C over night. The glutaraldehyde was removed and the disks were washed two times for 10 min with PBS and dehydrated in a series of ethanol (50–100%). Samples were dried with hexamethyldisilazane and subsequently gold-coated using a gold sputtering unit.

### Biofilm Quantification

Biofilm formation on the materials was analyzed using crystal violet staining as described before (Kwasny and Opperman, 2010). In brief, 2 ml of the bacterial suspension containing 2 × 10^8^ CFU were incubated on the materials for 24 h. Subsequently, supernatants were removed and the disks were washed three times with double-distilled water to remove non- or loosely adherent bacteria. For heat fixation, the plates were incubated at 60°C for 1 h. 200 μl of 0.06% crystal violet were added into each well and incubated for 5 min. The crystal violet was removed and the disks were washed three times with double-distilled water. Generated biofilms on the disks were eluted with 200 μl of 30% acetic acid. Photometric measurement of supernatants was performed at 600 nm in a multilabel microtiter plate (FLUOstar Omega, BMG Labtech).

### Material Eluate Test

A material eluate test was performed to analyze the impact of diffusing substances, released by the materials, on the bacterial biofilm formation. Four disks of each material were incubated in 6 ml TSB or LB broth for 24 h. 2 × 10^8^ CFU were resuspended either in undiluted or diluted (1:2) eluate broth of each material. 200 μl of the suspensions were added in a 96-well plate as triplicates and incubated for 24 and 48 h. Following incubation, the measurement of biofilm formation was carried out with crystal violet staining as described before. Tmx was used as reference and normalized to 100%.

### Statistics

Statistical analysis was performed using GraphPad Prism 5.0 (GraphPad Software, La Jolla, CA, United States). Values are expressed as mean + SE_*M*__*ean*_. Normal distribution was tested using the D’Agostino and Pearson Omnibus Normality Test. Non-normally distributed samples were compared using the Kruskal–Wallis test followed by a Dunn’s *post-hoc* test. For all statistical analyses, *p* < 0.05 were considered significant. ^∗^ to tmx: ^∗^*p* < 0.05, ^∗∗^*p* < 0.01, ^∗∗∗^*p* < 0.001; ^#^ to PMMA-MPC: ^#^*p* < 0.05, ^##^*p* < 0.01, ^###^*p* < 0.001; ^†^ to PSU: ^†^*p* < 0.05, ^†⁣†^*p* < 0.01, ^†⁣†⁣†^*p* < 0.001; ^+^ to PPSP: ^+^*p* < 0.05, ^++^*p* < 0.01, ^+++^*p* < 0.001. Tmx served as control material.

## Results

### Surface Attraction Properties of Liquid Silicon Rubbers (LSRs) Is Decreased Compared to Thermoplastic Polyurethane Materials

**FIGURE 2:**
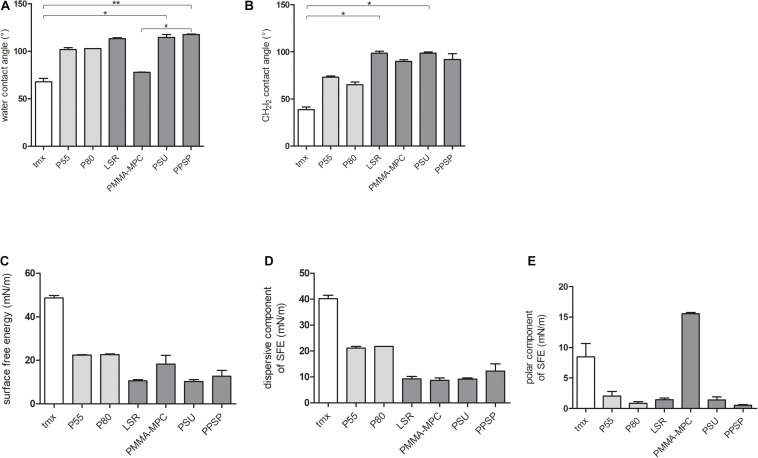
Surface attraction properties of liquid silicon rubbers (LSRs) is decreased compared to thermoplastic polyurethane materials. **(A)** water contact angles **(B)** CH_2_I_2_ contact angles **(C)** calculated SFE values **(D)** dispersive component of SFE and **(E)** polar component of SFE. Material surface characterization were carried out with *n* = 4. Data are represented as mean + SE_*Mean*_. Significances: * vs. tmx: * *p* < 0.05, ** *p* < 0.01, # vs. PMMA-MPC: # *p* < 0.05.

Using contact angle measurements of two liquids (deionized water and diiodomethane, CH_2_I_2_) with different densities and refractive indices, we determined the wetting properties and the surface free energies (SFE) as well as the polar and dispersive components of SFE of the materials to be tested according to the OWRK-method. With expectation of PMMA-MPC, contact angle measurement of deionized water on all TPUs and LSRs (P55, P80, LSR, PSU, PPSP) resulted in about one-third higher values (>100°) compared to the control tmx (Figure 2A). PMMA-MPC (∼75°) displayed comparable wettability to tmx. A water contact angle of a surface of >90° is referred as to be hydrophobic and <90° as hydrophilic. The contact angles of diiodomethane (CH_2_I_2_) on the tested materials were increased compared to tmx, with highest values for the LSR-based materials (Figure 2B). The calculated SFE of all tested materials was reduced compared to tmx. SFE values of P55, P80, and PMMA-MPC were comparable and up to three times higher compared to the unmodified LSR, PSU, and PPSP materials (Figure 2C). For the dispersive component of SFE, all LSR values were in a similar range and showed a significant reduction compared to the TPUs and the control (Figure 2D). The polar component of the SFE was significantly reduced in all materials except PMMA-MPC. Remarkably, the polar component of PMMA-MPC was more than 14 times higher than that of TPUs and other LSRs and twice that of tmx (Figure 2E).

### Cell Surface Hydrophobicity Between *S. aureus* and *S. epidermidis* Does Not Differ

The cell surface hydrophobicity was determined by the MATH/BATH (Microbial Adhesion to Hydrocarbons/Bacterial Adherence to Hydrocarbons) assay. Comparison of the percent hydrophobicity of the cell surface of *S. aureus* and *S. epidermidis* revealed no differences. Both strains are classified as highly hydrophobic with cell surface hydrophobicity of *S. aureus* 79.35% (±5.52) and *S. epidermidis* 71.14% (±4.6).

### LSR Coated With Polymethylmethacrylate-2-Methacryloyloxyethyl Phosphorylcholine (PMMA-MPC) Suppresses *S. aureus* Colonialization

**FIGURE 3:**
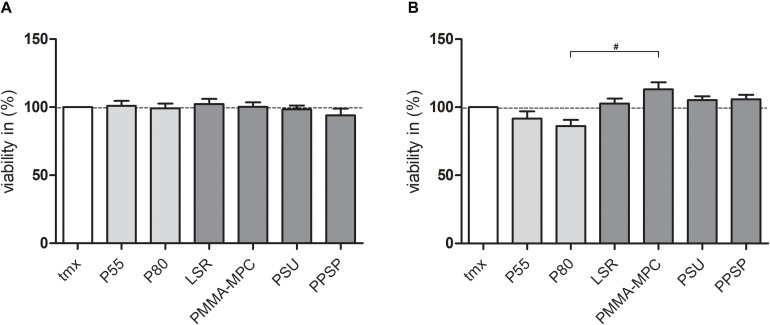
LSR coated with polymethylmethacrylate-2-methacryloyloxyethyl phosphorylcholine (PMMA-MPC) suppresses S. aureus colonialization. Materials were incubated with 1 × 10^8^ CFUs of **(A)** *S. aureus* and **(B)** *S. epidermidis* and bacterial viability was quantified after 1 h by WST assay. Values were normalized to tmx control (100%). Data are represented as mean + SE_*Mean*_ of three independent experiments carried out in duplicates. Significances: # vs. PMMA-MPC: # *p* < 0.05.

**FIGURE 4:**
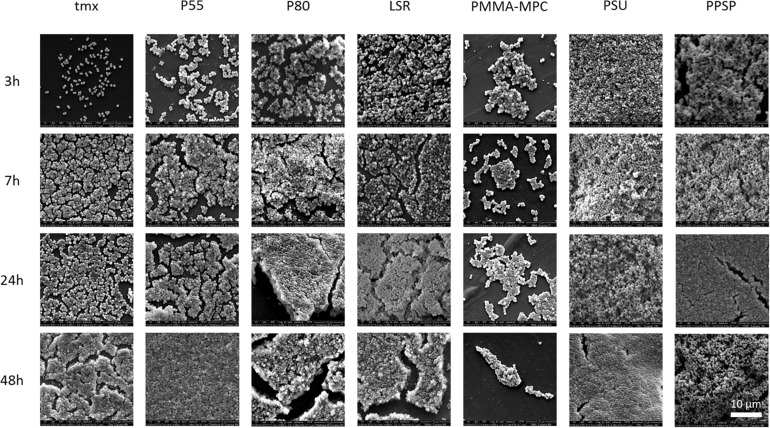
LSR coated with polymethylmethacrylate-2-methacryloyloxyethyl phosphorylcholine (PMMA-MPC) suppresses *S. aureus* colonialization. Materials were incubated with 2 × 10^8^ CFUs of *S. aureus* and colonialization was analyzed after 3, 7, 24, and 48 h by SEM. Magnification, ×4000.

**FIGURE 5:**
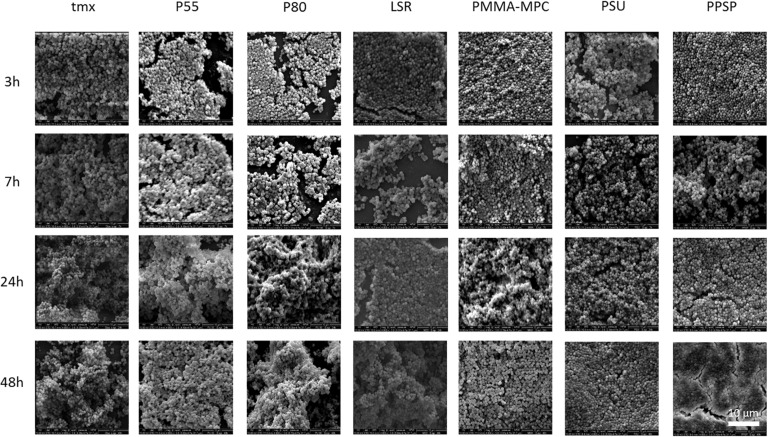
Investigated materials have no influence on *S. epidermidis* colonialization. Materials were incubated with 2 × 10^8^ CFUs of *S. epidermidis* and colonialization was analyzed after 3, 7, 24, and 48 h by SEM. Magnification, ×4000.

None of the tested materials had a negative effect on bacterial viability (Figure 3A). For *S. epidermidis* there was a significant increase in viability on PMMA-MPC in comparison to P80 (Figure 3B). The colonialization of the different bacterial strains on the material surfaces were analyzed by scanning electron microscopy (SEM). *S. aureus* was colonizing all tested materials after 7 h, with the only exception of PMMA-MPC. *S. aureus* failed to spread on the surface of PMMA-MPC (Figure 4). On PMMA-MPC, no confluent biolayer of *S. aureus* could be detected up to 48 h after seeding. In contrast, *S. epidermidis* covered almost all materials within 3 h (Figure 5).

### LSR Coated With Polymethylmethacrylate-2-Methacryloyloxyethyl Phosphorylcholine (PMMA-MPC) Significantly Reduces *S. aureus* Biofilm Formation

**FIGURE 6:**
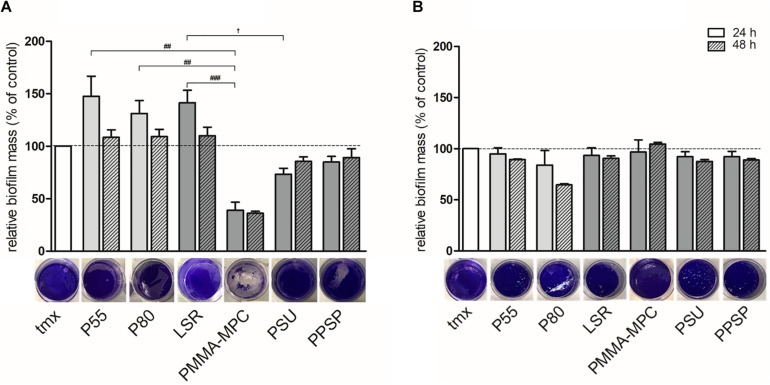
LSR coated with polymethylmethacrylate-2-methacryloyloxyethyl phosphorylcholine (PMMA-MPC) significantly reduces *S. aureus* biofilm formation. Materials were incubated with 2 × 10^8^ CFUs of **(A)** *S. aureus* and **(B)** *S. epidermidis* and biofilm formation was quantified after 24 h and 48 h by crystal violet staining. Representative pictures of biofilm formation stained with crystal violet after 24 h are shown. Values were normalized to tmx control (100%). Data are represented as mean + SE_*Mean*_ of three independent experiments carried out in duplicates. Significances: # vs. PMMA-MPC: ## *p* < 0.01, ### *p* < 0.001; ^†^ vs. PSU: ^†^ *p* < 0.05.

Bacterial biofilm formation was quantified by crystal violet staining. After 24 h, biofilm formation of *S. aureus* was enhanced on TPUs and unmodified LSR compared to tmx control (Figure 6A). In contrast, biofilm formation was reduced on modified LSRs. On PMMA-MPC biofilm formation of *S. aureus* was significantly reduced compared to TPUs and unmodified LSR (Figure 6A). For *S. epidermidis*, no pronounced differences were observed in terms of biofilm formation on the tested materials, except for a slight decrease on P80 (Figure 6B).

### Soluble Material Components Do Not Inhibit Biofilm Formation of *S. aureus*

**FIGURE 7:**
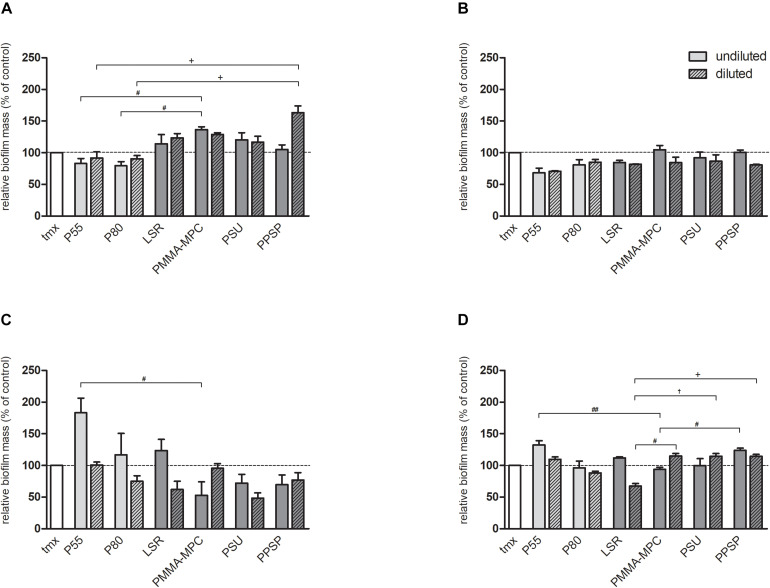
Soluble material components do not inhibit biofilm formation of S. aureus and S. epidermidis. 2 × 10^8^ cfu of **(A)** S. aureus 24 h and **(B)** *S. aureus* 48 h **(C)** *S. epidermidis* 24 h and **(D)** *S. epidermidis* 48 h were incubated with undiluted and diluted eluates from the materials and biofilm formation was quantified after 24 and 48 h by crystal violet staining. Values were normalized to tmx control (100%). Data are represented as mean + SE_*Mean*_ of three independent experiments carried out in duplicates. Significances: # vs. PMMA-MPC: # *p* < 0.05, ## *p* < 0.01; ^†^ vs. PSU: ^†^ *p* < 0.05; + vs. PPSP: + *p* < 0.05.

To investigate whether substances released from the materials can inhibit biofilm formation, an eluate test was performed. A marginal reduction in *S. aureus* biofilm formation after 24 h cultivation in undiluted or diluted eluate from TPUs compared to tmx was investigated (Figure 7A). Incubation with the eluate from the LSR samples showed increased biofilm formation compared to TPU samples and tmx after 24 h (Figure 7A) incubation time but not after 48 h (Figure 7B). This effect reached significance in the case of undiluted PMMA-MPC compared to P55 and P80 for 24 h (Figure 7A) and for diluted PPSP compared to P55 and P80. With regard to *S. epidermidis*, the eluates of the TPUs and the unmodified LSR caused an increase of biofilm formation after 24 h incubation with undiluted eluate broth compared to the other materials (Figure 7C). For 24 h biofilm formation of *S. epidermidis* with diluted eluate, a lower biomass production was observed for all materials, except for P55, compared to tmx. After 48 h, biomass production for P55, PMMA-MPC, PSU, PPSP was higher compared to the control tmx (Figure 7D). To summarize, *S. aureus* and *S. epidermidis* built biofilms in undiluted and diluted eluates. No eluate could prevent biofilm formation. However, selectively we observed a promoting or reducing effects on biofilm formation for individual materials, which in some cases, e.g., undiluted P55 compared to PMMA-MPC (Figures 7C,D) reached significance.

## Discussion

In the present study, two different commonly used TPUs (P55, P80), unmodified LSR and three modified LSRs (PMMA-MPC, PSU, PPSP) were evaluated for their anti-bacterial potential. We were able to demonstrate that the modified LSR with PMMA-MPC has a high resistance to *Staphylococcus aureus* adherence and biofilm formation. Moreover, we provide strong evidence that the inhibition of *S. aureus* biofilm formation was due to an anti-adhesive effect of the material surface.

The SFE is an essential measure of the wettability of solid surfaces. Several reports have shown that changes in the SFE of a material influence bacterial adhesion and biofilm formation (Busscher et al., 1986; Sinde and Carballo, 2000; Pereni et al., 2006). Low SFE results in weaker molecular attraction, whereas high SFE in general means strong attraction (von Fraunhofer, 2012). Lower attractive forces are accompanied by reduced attachment of bacteria and consequently lower biofilm formation. In our study, the SFE of all materials was reduced to varying degrees compared to tmx (Figure 2C). For the dispersive component of SFE, the LSR samples were all below the values of the control tmx and the two TPUs, P55 and P80 (Figure 2D). For the polar component of SFE, PMMA-MPC stood out with a significant increase (Figure 2E).

Since we can exclude bacterial cell-damaging effects of the material neither by direct material contact nor by the eluate based on the present results (Figures 3, 7), we attribute the observed anti-infectious effect to the polar fraction of the SFE of PMMA-MPC. Contradicting Harnett et al. (2007) reported that the polar component of the surface energy lower than 5 mN m^–1^ led to reduced cell spreading and that greater than 15 mN m^–1^ promoted spreading. In turn, Yuan et al. (2017) reported considerable bacterial adhesion of *E. coli* to polymer substrates despite of a low polar component of the surface energy. In addition, in the same study an air plasma treated material with the highest polar component (31.3 mN m^–1^) showed low bacterial adhesion. This suggests that the underlying mechanisms are very complex and can vary greatly in individual cases. Therefore, there is a need for further research on the relationship between bacterial adhesion and the components of SFE.

The observed effect of increased biomass on most materials within the first 24 h and then decreases after 48 h can be explained by the dispersion of the mature biofilm at later time points. The re-detachment of planktonic cells is a well-described process during the maturation of biofilms (Lister and Horswill, 2014; Büttner et al., 2015; Rabin et al., 2015). Both *S. aureus* and *S. epidermidis* seem to form a robust biofilm up to 24 h, which then appears to dissolve at further time intervals. This effect is more pronounced for *S. aureus* and partially reversed for *S. epidermidis*, especially for the undiluted eluate of the modified LSR samples (Figure 5B). The differences seen in bacterial adherence and biofilm formation of *S. aureus* and *S. epidermidis* could be due to the significant increase of *S. epidermidis* viability, at least for PMMA-MPC. These results are also in line with previous findings which highlighted that biofilm formation of *S. aureus* tends to be slower than that of *S. epidermidis* (Arciola et al., 2001; Cucarella et al., 2001; Vasudevan et al., 2003). In addition, *S. epidermidis* ATCC35984 is known to be a strong slime-producing strain (Wu et al., 2003; Oliveira et al., 2018) and therefore has better adhesion abilities due to the self-produced matrix proteins. While *S. aureus* produces a variety of toxins and adherence factors, production of EPS (extracellular polymeric substances) represents the main virulence factor of *S. epidermidis* on biomaterials. The EPS is composed of polysaccharides, proteins, nucleic acids, lipids and other biological macromolecules (Wingender et al., 1999). Both species produce poly-(1,6)-N-acetyl-D-glucosamine (PNAG), a surface polysaccharide as a main component and teichoic acid and eDNA (Jabbouri and Sadovskaya, 2010; Hou et al., 2012). Others (Izano et al., 2008; Flemming and Wingender, 2010) suggest that PNAG is a major matrix adhesin in *S. epidermidis* biofilms and a minor component of *S. aureus* biofilms and that eDNA is a major structural component in the *S. aureus* biofilm matrix and a minor structural component in the *S. epidermidis* biofilm matrix. The composition and numbers of EPS components influence interaction with substrate surfaces. This was investigated in a previous study on different *S. epidermidis* and *S. aureus* strains, making *S. epidermidis* more receptive to a cationic and amphiphilic polymer in this case (Rawlinson et al., 2011). In addition, more hydrophobic cells adhere to hydrophobic surfaces, while hydrophilic cells prefer to adhere to hydrophilic surfaces (Kochkodan et al., 2008; Giaouris et al., 2009; Krasowska and Sigler, 2014). Both of our bacterial strains showed hydrophobic surface properties suggesting that this cannot be the reason for the different effects. Further studies on the composition of strain EPS are need to specify these differences. The fact that a species can show divergent adhesion patterns, as shown by Di Ciccio et al. (2015) for 67 isolates of *S. aureus*, underlines that the adhesion of bacteria is not uniform and requires further research. In summary, this study presents new modified LSRs that achieved better results in terms of anti-biofilm formation strategies compared to the commonly used TPUs. PMMA-MPC proved to be the most promising modification. These findings are very important due to the risks of biofilm formation especially in the context of implant application. The need for innovative materials for future approaches in new medical fields such as cardiovascular implants is of great relevance also in regard to the field of antibiotic-resistant microorganisms (Li and Webster, 2018). In this context, it would be of further interest to determine whether (1) the additional modification of PSU and PPSP with zwitterionic molecules and (2) the use of this materials in other innovative approaches like nanostructured systems (Zhang et al., 2020; Linklater et al., 2021) lead to improved microbial defense. Another fact is that zwitterionic biomaterials have shown opposite behavior in inhibiting the adhesion of different bacterial strains. For future research, it would be beneficial to investigate this effect on other bacteria associated with biofilm-associated infections, e.g., *Streptrococcus* spp. or *Enterococcus* spp. (Jamal et al., 2018).

## Data Availability Statement

The datasets generated for this study are available on request to the corresponding author.

## Author Contributions

FW conceived and designed the analysis, collected the data, performed the analysis, and wrote the manuscript. MK performed the analysis and wrote the manuscript. KS and AB designed the materials and contributed data. AR, CM, and ER wrote the manuscript. SO, VS, and NG contributed data and analysis tools. MS conceived and designed the analysis, performed the analysis, and wrote the manuscript. All authors contributed to the article and approved the submitted version.

## Conflict of Interest

The authors declare that the research was conducted in the absence of any commercial or financial relationships that could be construed as a potential conflict of interest.
